# Promoter Hypermethylation of LATS1 Gene in Oral Squamous Cell Carcinoma (OSCC) among North Indian Population

**DOI:** 10.31557/APJCP.2021.22.3.977

**Published:** 2021-03

**Authors:** Harsh Goel, Runjhun Mathur, Saima Syeda, Anju Shrivastava, Abhimanyu Kumar Jha

**Affiliations:** 1 *Department of Biotechnology, Institute of Applied Medicines and Research, Ghaziabad, Uttar Pradesh, India. *; 2 *Dr. A.P.J. Abdul Kalam Technical University, Lucknow, Uttar Pradesh, India.*; 3 *Department of Zoology, Delhi University, India. *

**Keywords:** Oral squamous cell carcinoma (OSCC), epigenetic changes, LATS2 gene, promoter hypermethylation

## Abstract

**Background:**

*LATS1* (Large Tumor Suppressor, isoform 1) is a gene that forms a complex with the cyclin-dependent kinase, CDK1, and regulates cell cycle progression. Genetic modifications lead to a loss in the activity of *LATS1* gene. OSCC is the most commonly emerging cancer caused by genetic as well as epigenetic changes. Epigenetics changes vary from one population to another because these are influenced by dietary factors and environmental factors. Tobacco chewing and smoking has been reported as major risk factors in OSCC. No report was found in the previous literature showing promoter hypermethylation of *LATS1* gene.

**Methods:**

A total of 50 OSCC patients and 20 normal individuals were recruited in this study. Blood samples (50) from OSCC patients and blood samples (20) from healthy individuals as controls were used in the present study. Isolation of genomic DNA was carried out from blood using the standard phenol-chloroform extraction. Further Isolated DNA was modified with sodium bisulfite using the agarose bead method and finally, the methylation studies of *LATS1* gene were carried out using Methylation-Specific PCR (MSP-PCR).

**Results:**

19 out of 50 patients (38.0%) were found to be methylated for LATS1 gene.; a statistically significant result was obtained (p -value= < 0.05) with an odds ratio of 0.37 in cases compared to controls. The status of methylation of *LATS1* genes was also found to be statistically significantly associated with smokers and tobacco chewers (p-value = < 0.05). The methylation of* LATS1* gene showed a significant risk of developing OSCC in patients.

**Conclusion:**

These results suggest that the *LATS1* gene may provide a better alternative as a diagnostic biomarker. This is the first report on the promoter hypermethylation of *LATS1* gene in OSCC patients among the North Indian population.

## Introduction

Cancer is the most common, vicious, and dangerously increasing diseases of the world today, associated with high morbidity and mortality. Oral Squamous Cell Carcinoma (OSCC) is the sixth most frequent type of cancer observed in the Indian population. Survival rates of patients with advanced OSCC have not increased significantly in recent years (Al-Swiahb et al., 2010). Oral cancer is a blended interaction between Genetic, epigenetic, and environmental factors. Development of OSCC is a multistep process resulting due to chronic inflammation and Genetic factors such as changes in oncogenes and Tumor Suppressor Genes (Hanahan and Weinberg, 2011). Tobacco consumption and smoking is the leading cause of OSCC. The most important etiologic factors did not seem to be the sole cause of cancer. Epigenetic mechanisms could also contribute to the silencing of Tumor-Suppressor Genes. Epigenetics relates to the heritable alterations in Gene expression that do not result from changes in Gene nucleotide sequence. Epigenetic modifications play a crucial role in cell cycle regulation, and differentiation, along with these modifications, inhibit Genes from Genetic mutations (Jha et al., 2016). DNA methylation was the earliest epigenetic changes to be seen in cancer cells. It plays a significant role within the regulation of the Gene expression and chromatin design by providing a stable Gene silencing mechanism (Mahmood and Rabbani, 2019). Promoter Methylation induced Gene silencing leads to cancer initiation and progression. In mammals, DNA methylation is directly linked with the covalent modification of the cysteine residue within the CpG dinucleotides. Methylation regions rich in CpG promoters inhibit DNA transcription by altering the binding of histone complexes (Lu et al., 2006). The DNA methylation markers can be used in cancer diagnostics for both disease classification and detection because epigenetic changes occur very early in the oncogenic process (Ehrlich, 2019). A Tumor Suppressor Genes, when functioning normally, inhibit the growth of Tumors; losses of their regular functions (inactivation) are needed. For Tumorigenesis. Allelic failures, Gene variations, and cryptic deletions are usually recognized as mechanisms required for the inactivation of Tumor Suppressor Genes. Tumor Suppressor Genes can be classified into two groups: Genes that directly control Tumor growth by inhibiting cell proliferation or increasing cell death (“gatekeepers”) and genes whose inactivation causes genetic variability, which in turn leads to mutations that increase neoplasm growth (Hisaoka et al., 2020).

LATS1 (Large Tumor Suppressor Gene) is a Ser/Thr kinase associated with the Tumor Suppressor pathway called the Hippo-LATS/Warts pathway that represses tumor outgrowth by controlling cell proliferation, cell growth, and cell death (Cao et al., 2020). LATS1 Gene is linked with mitotic events in mammalian cells, and that loss of its function interrupts normal cell cycle regulation, leading to the development of Tumors (Chan et al., 2005). In mammalian cells, LATS1 Gene interacting with Cdc2 in the mitotic phase and negatively regulates Cdc2 activity (Hao et al., 2008). LATS1 plays a vital role in controlling cell fate by promoting the degradation of YAP/TAZ proteins by phosphorylation-mediated ubiquitination and regulating a tumor-suppressive transcriptional factor p53, cell cycle checkpoint regulators, and the centrosomal protein phosphatase CDC25B (Nozaki et al., 2019). Overexpression and demethylation of the LATS1 gene inhibited cell proliferation, downregulated YAP expression, induced cell apoptosis, and cell cycle arrest in renal cell carcinoma cells (Williams and Stoeber 2012). LATS1 phosphorylates angiomotin to suppress cytokinesis, cell migration, and angiogenesis by inhibiting F-actin binding and polymerization via negative modulation of LIMK1 (Dai et al., 2013; Yang et al., 2006). LATS1 acts as a Tumor Suppressor Gene Promoter methylation targeting LATS1 inactivation in mice leads to the development of many tumors, such as ovarian cancer and sarcomas (St John et al., 1999). Cytoplasmic LATS1 gene expression was positively correlated with lymph node metastasis (Luo et al., 2020). LATS1 is downregulated in several human cancers such as astrocytoma, colorectal cancer, lung cancer, breast cancer, and glioma due to hypermethylation. LATS1 overexpression not only significantly inhibits migration, invasion cell growth and also delays cell cycle progression from G2/M to G1 in vitro glioma U251 cells (Ji et al., 2012). LATS1 knockout mice showed the development of mitotic defects, chromosomal misalignment, mouse embryonic fibroblasts (MEFs), multipolar spindle formation, centrosomal overduplication, cytokinesis failure, and chromosomal bridging (Wu et al., 2018). It was shown that LATS1 overexpression with upregulated p27 expression stimulated YAP phosphorylation inhibited cell proliferation in cervical cancer cells (Deng et al., 2017). The malignant transformation was strongly correlated with low membrane expression of LATS1 in the cervical epithelium (Zhou et al., 2014). LATS1 overexpression was observed to repress growth, induce apoptosis, and mesenchymal to epithelial transition (MET). Changes in the LATS1 function might be of pathological significance in human Tumorigenesis (Kamikubo et al., 2003). Based on the above background, the objectives of this study were to estimate the promoter hypermethylation status of *LATS1* genes in circulating blood of OSCC patients and to determine their association with clinicopathological features of OSCC.

## Materials and Methods

*Sample Collection*

Blood samples (50) were collected with the informed consent of patients diagnosed with OSCC after taking the necessary ethical clearance from Dharamshila Cancer Hospital & Research Centre, New Delhi. The blood samples (20) from healthy individuals (as controls) were also obtained. Only patients diagnosed with OSCC were considered in the patients group, while individuals not suffering from any cancer were considered as controls. The samples were further used for DNA extraction.

*DNA Exraction*

The DNA isolation was carried out from blood as well as serum samples. One part of this study was carried out on blood samples, and for the other part of this study, serum was separated from the blood by centrifuging at 2,200 rpm. Leucocyte or White Blood Cells (WBCs) collected from blood samples were lysed in digestion buffer (10 mM Tris-HCl, pH 8.0, 10 mM EDTA, 150 mM NaCl and 2% SDS) containing proteinase K (0.2 mg/ml). DNA was then purified using the standard phenol-chloroform extraction and ethanol precipitation (Hoque et al., 2004).

*Sodium Bisulfite Modification*

This method allows the particular study of methylation in a specific region by converting all non-methylated cytosine into uracil, while methylated cytosines remain unchanged. Isolated DNA was modified with sodium bisulfite using the agarose bead method (Tiwari et al., 2009).

*Purity of DNA*

To quantify the amount of DNA, Nanodrop ND-1000 spectral photometer was used. The quality of DNA was checked using agarose gel electrophoresis.

*Methylation-Specific PCR (MS-PCR)*

The methylation status of the *LATS1* gene was detected using MS-PCR assay for bisulfite-converted DNA (Herman et al., 1996). The amplification of the bisulfite-treated DNA was done through methylation-specific – PCR; MS-PCR was carried out using specific primers for methylation and unmethylation for the *LATS1* genes (Table 1). MSP was performed to check the promoter hypermethylation of the *LATS1* gene in OSCC patients (Figure 2). Photomicrograph representing 2.5% agarose gel electrophoresis of PCR amplified *LATS1* gene that was carried out in 1X TAE buffer at a voltage supply of 80 V. 

The following steps and temperature conditions were used in the process: the initial denaturation was carried out at 95°C for 10 mins. The PCR cycles consisting of denaturation at 95°C for 40 sec, annealing temperature for LATS1: M = 55°C, U =56°C for 40 sec and elongation step was done at 72°C for 40 sec for 35 cycles. The final extension was carried out at 72°C for 10 min.

*Statistical Analysis*

The association between the hypermethylation of the* LATS1* gene and risk of OSCC was estimated by computing odds ratios (ORs) and 95% confidence intervals (CI) using the Chi-square test which included several potential confounding variables. Statistical analysis was performed using Stat Calc (Statistical Calculator) of Epiinfo tool version 7.2. P<0.05 was considered to indicate a statistically significant difference. 

## Results

*Isolation of DNA*

**Figure 1:**
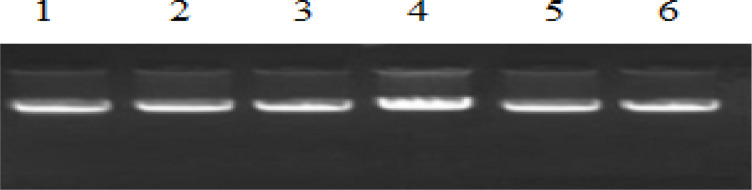
Qualitative Analysis of DNA (Isolated DNA from Blood Samples of OSCC Patients and Controls). Lane No- 1-4, DNA of OSCC patients; Lane No- 5-6, DNA of healthy individuals (controls).

The DNA isolated from the blood and serum samples were run on the 1% agarose gel and visualized under the gel documentation unit. This was carried out to check the quality of DNA (Figure 1). The isolated DNA was also quantified using Nanodrop ND-1000 spectral photometer.

*MS PCR Result of LATS1 GENE*

**Figure 2:**
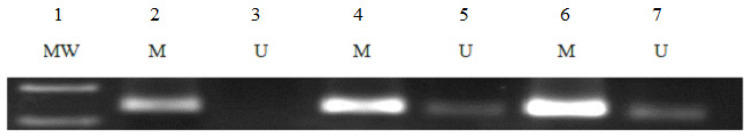
MSB (Methylation Specific Band) and UMSB (Unmethylation Specific Band) of LATS1 in Blood Samples of OSCC Patients. Lane No- 1, Molecular Marker 100 base pair; Lane No- 2,4,6, MSB (Methylation Specific Band) in the blood samples of OSCC patients; Lane No-3,5,7, UMSB (Unmethylation Specific Band) in the blood samples of OSCC patients

**Figure 3:**
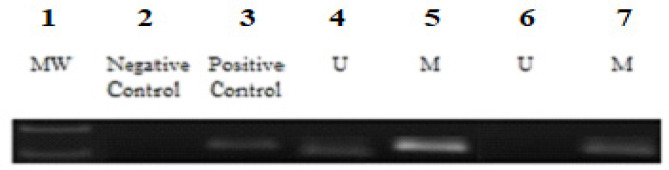
Methylation-Specific PCR Including Negative Control and Positive Control. Lane No- 1, Molecular marker 100 base pair; Lane No- 2, Negative control (H_2_O); Lane No-3, positive control (in vitro methylation)

Methylation Specific PCR was performed to check the promoter hypermethylation of *LATS1* Gene in OSCC patients among the North Indian population (Figure 2) and (Figure 3).

*Status of promoter hypermethylation*

**Figure 4:**
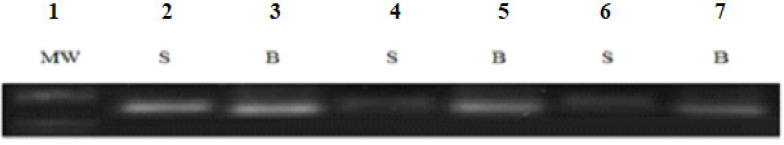
Comparision of the Methylation Status *LATS1* Gene in Blood and Serum Samples of OSCC Patients. MSB (Methylation Specific Band) of LATS1 in serum and paired blood samples of OSCC patients along with ladder (Size 138 bp). Lane No- 1: Molecular marker 100 base pair; Lane No- 2,4,6, MSB (Methylation Specific Band) in serum samples of OSCC patients. Lane No- 3,5,7, MSB (Methylation Specific Band) in paired blood samples of OSCC patients

Hypermethylation of LATS1 was observed in 38% of OSCC patients. 19 out of 50 samples were found to be promoter hypermethylated through MSP (Methylation Specific PCR) in OSCC patients (Figure 2). The statistical analysis of promoter hypermethylation of *LATS1* Gene was found to be significant (p-value= 0.017) with an odds ratio of 0.37 (Table 2). Hypermethylation of the *LATS1* gene was observed in Serum samples, although the intensity of MSB was found to be less in serum samples (Figure 4).

*Correlation between Clinicopathological Information and Methylation Status in OSCC*

In this study total 50 samples were taken, out of which 30 were tobacco chewers and similarly in case of smokers 30 samples were taken and out of which 20 were smokers. The methylation of *LATS1* gene showed a significant risk of developing OSCC in patients (p-value<0.05) (Table 2). LATS1 was observed to be methylated 50% in tobacco chewers and 66.6% in smokers among patients. It was not observed among controls. The hypermethylation of the promoter region of the *LATS1 *gene was also found to be statistically significant in tobacco chewers (Table 3) and smokers (Table 4) as the p-value was found to be < 0.05.

**Table 1:** Sequence of the Primers for* LATS1* Gene

Name of Gene	Sequences (5′–3′)	Annealing tem (°C)	Product size
*LATS1 M*	F: GGAGTTT CGTTTTGTC	55 °C	138 bp
	R: CGACGTAATAACG AACGCCTA		
*LATS1 U*	F: TAGGTTGGAGTGTGGTGGT	56 °C	121 bp
	R: CCCAACATAATAACAAACACCT		

M, Methylated; U, Unmethylated

**Table 2:** Frequency of Methylation and Unmethylation of *LATS1* Gene with the Relative Risk of OSCC in Patients and Healthy Controls

Gene	Methylation status	Cases (n=50)	Control (n=20)	OR	95%CI	P-value
*LATS1*	M	19 (38.00 %)	0 (00.00 %)	0.37	0.16–0.84	0.017
	U (ref)	31 (62.00 %)	20 (100.00 %)			

OR, odds ratio; 95% CI, 95 % confidence interval; ref, reference

## Discussion

Oral squamous cell carcinoma (OSCC) is one of the most popular types of oral neoplasm, accounting for over 90 % of all mouth fatalities and 38 % of head and neck neoplasms. In fact, despite advances in the area of oral cancer detection, prevention, and multimodality approaches, the overall 5-year survival for OSCC remains to be modest at best. The common significant factor influencing OSCC survival after treatment is the grade of the Tumor at diagnosis. Hence, to promote long-term results, immediate detection in combined with primary and secondary control strategies is critical. A methyl group is covalently joined to cytosine C5. When DNA is treated with bisulfite, unmethylated cytosines are converted to uracil, but methylated cytosines are protected. In this phenomenon, the promoter region of the gene is hypermethylated which causes global hypomethylation to the gene. When these methyl groups are segregated from the cytosine nucleotide then the expression of the sequence is altered. This is called a reversal of methylation. This causes the expression to express which is delayed due to methylation. Epigenetic changes vary from population to population as they are mainly dependent upon the environmental and dietary factors which differ greatly (Jha et al., 2017).

Large Tumor Suppressor gene 1 (*LATS1*) are key tumor suppressor genes in the cell cycle regulation and DDR signaling (Najafi et al., 2016). Previous studies have indicated the promoter methylation profiles of P14ARF, MGMT, CDH1, APC, ATM, P15INK4b, P16INK4a, FADD, FAS, ERK and RAF1 in OSCC (Kordi-Tamandani et al., 2010b; Rigi-Ladiz et al., 2011; Kordi-Tamandani et al., 2012; Saberi et al., 2014; Kordi-Tamandani et al., 2014). To the best of our knowledge, no published work is there from India on the promoter hypermethylation of the selected gene. So, the present research also focused on correlating the methylation status of *LATS2* gene with these risk factors like tobacco chewing and smoking in OSCC patients among the North Indian population. In the present study, the DNA was isolated from the collected blood samples of a cancer patient (OSCC) and control samples (patients not suffering from cancer). Moreover, isolation of DNA was followed by sodium bisulfite modification by agarose bead method and E Z DNA Gold Methylation Kit (Zymo Research, US), after which MSP was performed. A statistically significant difference in methylation of LATS2 (p-value0.05) between patient and control samples was observed. The methylation of LATS2 showed the risk of developing OSCC in patients. It has been proved that the outcome novel methylation markers may be used for designing drugs that modify methylation statuses in OSCC (Mikeska and Craig, 2014.) Hence, based upon the present study LATS2 could be used as a diagnostic biomarker in OSCC patients among north Indian population. Investigations on broader sample size are required to declare the clinical applicability of LATS2 hypermethylation in larger groups of patients. Early estimation of LATS2 hypermethylation might allow the identification of subgroups of patients with poor diagnosis, who might require a diverse therapeutic strategy. Therefore, a future study needs to be carried out to explore the status of this potential biomarker in the clinical control of OSCC and to estimate whether it can contribute to personalized treatment strategies.

*Conclusion and Future Perspectives*

Promoter methylation of Tumor Suppressor genes is a critical factor in the carcinogenesis of OSCC.The Study of DNA methylation is a beneficial procedure for evaluating the biological characteristics of oral cancers and may be a useful diagnostic biomarker. Hence, the present study was intended to investigate the methylation status of *LATS2* gene and to correlate the methylation status of these genes with the risk of OSCC statistically. The risk of OSCC was also calculated and was found to be significant. This confirms that LATS2 hypermethylation is an important step in the OSCC patients among north India population and it can probably be used as a diagnostic biomarker in OSCC patients. Further the study on reversal of hypermethyation of *LATS 2* gene and its reactivation needs to be carried out and can provide a good breakthrough in the study involving cancer therapy. Thus research needs to be carried out on a large scale as the sample size in the present study was small to draw any complete statistical conclusion. However this study is very important as this is the first study on the methylation status of this gene in OSCC among the North Indian population. 
